# Endoplasmic Reticulum Subproteome Analysis Reveals Underlying Defense Mechanisms of Wheat Seedling Leaves under Salt Stress

**DOI:** 10.3390/ijms22094840

**Published:** 2021-05-03

**Authors:** Junwei Zhang, Dongmiao Liu, Dong Zhu, Nannan Liu, Yueming Yan

**Affiliations:** College of Life Science, Capital Normal University, Beijing 100048, China; zhangjunwei_22@163.com (J.Z.); liudongmiao1230@163.com (D.L.); ZD19920804@163.com (D.Z.); liunnCNU@163.com (N.L.)

**Keywords:** wheat, salt stress, physiological characteristics, ER proteome, label-free quantitation

## Abstract

Salt stress is the second most important abiotic stress factor in the world, which seriously affects crop growth, development and grain production. In this study, we performed the first integrated physiological and endoplasmic reticulum (ER) proteome analysis of wheat seedling leaves under salt stress using a label-free-based quantitative proteomic approach. Salt stress caused significant decrease in seedling height, root length, relative water content and chlorophyll content of wheat seedling leaves, indicating that wheat seedling growth was significantly inhibited under salt stress. The ER proteome analysis identified 233 ER-localized differentially accumulated proteins (DAPs) in response to salt stress, including 202 upregulated and 31 downregulated proteins. The upregulated proteins were mainly involved in the oxidation-reduction process, transmembrane transport, the carboxylic acid metabolic process, stress response, the arbohydrate metabolic process and proteolysis, while the downregulated proteins mainly participated in the metabolic process, biological regulation and the cellular process. In particular, salt stress induced significant upregulation of protein disulfide isomerase-like proteins and heat shock proteins and significant downregulation of ribosomal protein abundance. Further transcript expression analysis revealed that half of the detected DAP genes showed a consistent pattern with their protein levels under salt stress. A putative metabolic pathway of ER subproteome of wheat seedling leaves in response to salt stress was proposed, which reveals the potential roles of wheat ER proteome in salt stress response and defense.

## 1. Introduction

As one of the three major crops in the world, wheat (*Triticum aestivum* L.) is a staple source of food and protein in the human diet. However, global food security is facing great challenges, including arable land reduction, extreme climate change, and environmental pollution. Plants are often exposed to a variety of abiotic stress environments, among which salt stress significantly impacts crop growth and yield formation. High salt levels affect an estimated 20% of arable land, globally. [1]. The expansion of saline soil damages crop production at the cost of US $27.3 billion annually seriously threatens the world’s human populations [2,3].

The most obvious effect of salt injury on plants includes the decreased growth rate of new leaves that is proportional to the osmotic pressure of roots. The high salt content in leaves is a direct cause of plant death [4]. The primary impacts of elevated ionic strength are osmotic stress and ion toxicity. Osmotic stress first causes plant hormone abscisic acid to accumulate, leading to a series of adaptive responses [5]. Excessive sodium ion leads to ion toxicity and triggers a series of reactions that further activate the expression of *ENA1* and other target genes. *ENA1* encodes Na^+^-ATPase formation, urging the cell to pump excess sodium ions out of the cell, and finally restoring the ion homeostasis inside and outside the cell. Plants use calcium-dependent protein kinase pathways, also known as salt hypersensitivity pathways or salt overly sensitive (SOS) pathways for salt stress signal transduction [5]. In Arabidopsis, research has focused on the interaction and ion homeostasis mechanism of the three main proteins of the SOS pathways SOS1, SOS2 and SOS3 [6].

As an important organelle unique to eukaryotes, endoplasmic reticulum (ER) can form into tubules, vesicles and tubular interconnected reticular membrane systems in the cells. There are two kinds of ER present that are distinct in structural features: rough ER and smooth ER. The rough ER is involved in protein synthesis, processing and transport, while smooth ER participates in synthesizing lipids, phospholipids and steroids [7]. When subjected to endogenous and exogenous stresses, the accumulation of the misfolded proteins activates an ER stress response in which cells produce a series of responses to achieve a new ER homeostasis. One can detect the misfolded proteins using an ER quality control (ERQC) system and degrade them through an ER-associated degradation (ERAD) system. Plant ER stress response is an important mechanism for abiotic stress defense, which enhances stress resistance in plants and alleviates damages that various stresses cause [8]. The ER plays the role of protein factory and calcium bank, and it is an environment for protein quality monitoring mediated by various signal pathways and protein folding auxiliaries. Unfolded or misfolded proteins accumulate under adverse environmental conditions and leads to ER stress and cell death [9].

Generally, proteomics is a powerful approach performed at the large scale level while currently enabling also high-throughput procedures in an automatic and therefore more precise and safe manner [10,11]. Among different goals, proteomics also enables one to provide insight concerning plant responses and defense mechanisms under various biotic and abiotic stresses [12]. In recent years there has been great progress in plant proteomics, and subcellular proteomics research has become an important field to decipher plant cell responses during development and upon exposure to environmental stresses [13]. To date as of 2021, researchers have performed subcellular proteomic analyses in various plant species, such as soybean cell wall proteome, in response to flooding stress [14]; chloroplast proteome has been involved in photosynthesis and abiotic stress in rice and mangrove [15,16]; mitochondrial proteome in Arabidopsis tissues [17]; and finally, soybean plasma membrane proteome in response to osmotic stress [18]. In terms of plant ER subproteome studies, there has been relatively little work. The authors of [19] identified the flooding response proteins rich in ER by using non-gel and 1D gel-based proteomic techniques, finding that flood stress mainly affected the protein synthesis and glycosylation function of ER in soybean root tips. Through a gel-free/label-free proteomic approach, [20] found that the increase of cellular solute calcium levels induced by drought and flood stress might disturb the ER environment and affect protein folding in soybean root tips. In wheat, the subcellular proteomic studies are mainly performed in chloroplast [21,22,23], mitochondria [24,25] and plasma membrane [26]. This is because these subcellular structures are relatively easy to isolate intact and remove contamination of proteins from other organelles. However, other subcellular structures that are diffuse and amorphic such as ER and Golgi apparatus are very difficult to isolate and characterize. Concurrent to the development of subcellular proteomics, powerful methods have been established to effectively isolate the ER and characterize its proteome. These include various hydrodynamic techniques such as differential centrifugation and density gradient centrifugation. In more recent studies, purity has been assessed via electron microscopy and/or Western blotting for known ER-located proteins using calreticulin, BiP and calnexin as common ER markers. Considering that wheat has a huge genome and proteome, isolating and purifying wheat ER components face a significant challenge. Therefore, the ER proteome characteristics in wheat and its roles in abiotic stress response remain unclear.

Along with the development of subcellular proteomics, some powerful methods have been established to effectively isolate ER and characterize ER proteome such as ultracentrifugation combined with differential centrifugation and density gradient centrifugation [27]. In the present work, we performed the first ER proteomic analysis of wheat seedling leaves under salt stress using a label-free quantitative proteome approach. We aimed to reveal the ER proteome profiling and the potential roles of the key ER proteins involved in salt stress response. Our results provided new insights into the underlying mechanisms of plant subcellular organelles in response to abiotic stress.

## 2. Results

### 2.1. Phenotype and Physiological Changes of Wheat Seedlings under Salt Stress

**Figure 1:**
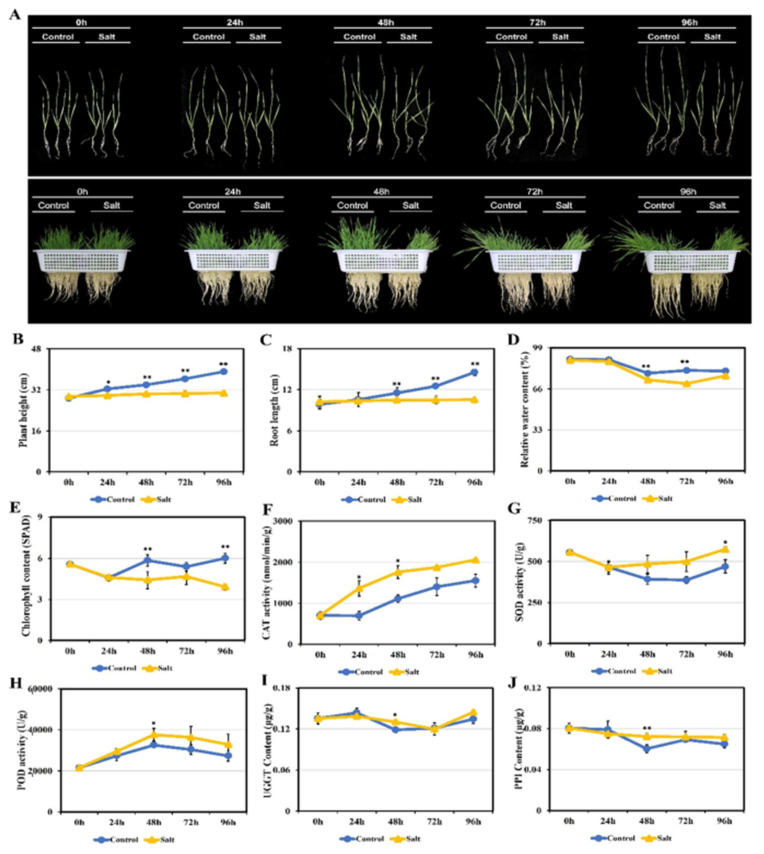
Morphological and physiological changes of wheat cultivar Zhongmai 175 seedling leaves under normal and salt stress conditions. (**A**) Morphology of individual seedling and bunch seedling. (**B**) Plant height. (**C**) Root length. (**D**) Relative water content. (**E**) Chlorophyll content. (**F**) CAT activity. (**G**) SOD activity. (**H**) POD activity. (**I**) UGGT content. (**J**) PPI content. Statistically significant differences were calculated based on an independent Student’s *t*-test: * *p* < 0.05; ** *p* < 0.01.

The morphological and physiological characteristics of wheat seedlings showed obvious changes under salt stress (Figure 1). Compared to the control group, the seedlings experienced leaf curl, lodging, wilting and yellowing with the increase of salt stress time, but particularly after 96 h of salt treatment (Figure 1A). Seedling height and root length significantly decreased (Figure 1B–C), indicating that salt stress severely inhibited plant growth.

Salt stress also significantly altered the physiological characteristics of seedling leaves. Compared with the control group, both relative water content and chlorophyll content gradually decreased under salt stress, though there was more significant reduction at 48–96 h (Figure 1D,E). On the contrary, the activities of catalase (CAT), superoxide dismutase (SOD) and peroxidase (POD), which are involved in plant reactive oxygen species (ROS) scavenging, significantly increased while spending time under salt stress (Figure 1F–H). Plants usually produce significant amounts of ROS in the wake of salt stress, and the increased activities of these enzymes enhance plant stress tolerance through a ROS scavenging mechanism. Meanwhile, UDP-glycosyltransferase and peptidyl prolyl cis-trans isomerase activities are closely related to ER stress response; this also increased after salt treatment, particularly at 48 h (Figure 1I,J). The process may promote plant salt tolerance through accelerating the misfolded proteins’ degradation.

### 2.2. Quality Assessment of the Isolated ER Proteome

**Figure 2:**
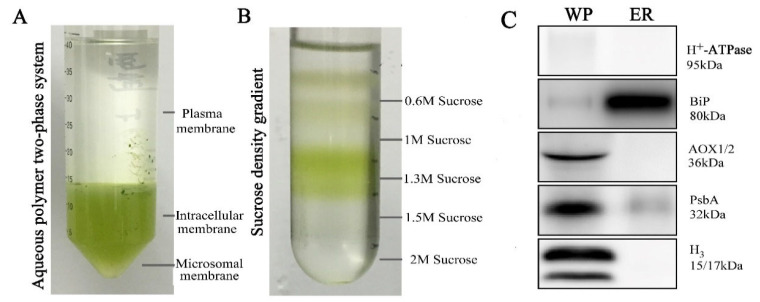
Purity assessment and immunoblotting analysis of the extracted ER proteins. (**A**) Discontinuous Percoll gradient. (**B**) Morphological observation of isolated chloroplasts. (**C**) Immunoblotting analysis using antibodies against plant cell compartment markers, including H+-ATPase (Plasma membrane), BiP (Endoplasmic reticulum), AOX1/2 (Mitochondrion), PsbA (Chloroplast) and anti-H3 (Nuclear). WP: whole protein of wheat leaves.

In this study, researchers extracted the components of intima media except plasma membrane and Golgi apparatus from lower layers through two phase separation systems (aqueous polymer phase), exhibited in Figure 2A. ER was further differentiated from other endometrial systems through a series of sucrose density gradients in 0.6–2 M. The sucrose density gradient between 0.6 M and 1.3 M was endoplasmic reticulum (Figure 2B). To test the purity of ER proteins, we used five polyclonal antibodies against different organelle-specific proteins using Western blotting, including plasma membrane specific protein antibody H^+^-ATPase, ER specific protein antibody BiP, mitochondrial specific protein antibody AOX1/2, chloroplast specific protein antibody PsbA and nuclear specific protein antibody H3. As shown in Figure 2C, one extremely strong ER-specific protein BiP band was observed when compared against leaf ER proteome, indicating that the ER protein was effectively enriched. The remaining four organelle-specific protein antibodies had no obvious signals (Figure 2C). These results demonstrated that the ER extracted proteins from wheat leaves show high purity without clear protein contaminations from other organelles.

### 2.3. Salt Stress Responsive DAPs in ER Subproteome and Subcellular Localization

**Figure 3:**
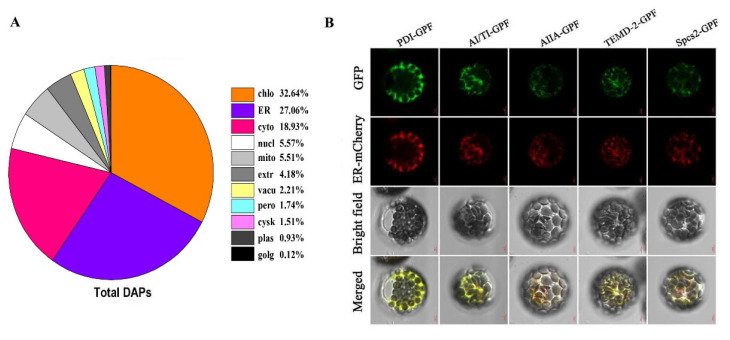
Subcellular localization of the proteins identified in wheat ER proteome under salt stresses. (**A**) Subcellular localization prediction of total proteins identified in the wheat endoplasmic reticulum. Chlo, chloroplast; ER, endoplasmic reticulum; Cyto, cytoplasm; Nucl, nucleus; Mito, mitochondria; Pero, peroxisome; Plas, plasma membrane; Extr, extracellular; Cysk, cytoskeleton; Vacu, vacuole. (**B**) Subcellular localization assay of five reprehensive proteins by wheat protoplast transformation. GFP, green fluorescent protein; ER-cherry; bright field, the field of bright light; merged, merged GFP fluorescence; ER-cherry, and the field of bright light; 16,318, empty vector; PDI: protein disulfide-isomerase; AI/TI: alpha-amylase/trypsin inhibitor; AIIA: probable ureidoglycolate hydrolase; TMED2:GOLD domain-containing protein; Spcs2: probable signal peptidase complex subunit 2; bar = 5 μm.

Label-free quantitative ER proteome analysis of wheat seedling leaves under normal conditions and salt stress identified 34,732 peptides corresponding to 6663 unique proteins with a high confidence (Table S1 and S2). Among them, salt stress changed the expression of 968 proteins by more than double compared with the control. Of these proteins, 516 were upregulated and 452 were downregulated (Table S3). In particular, 107 ribosomal proteins were identified, including 1 upregulated protein and 106 downregulated proteins. The subcellular localization prediction of the other 861 DAPs showed that there were 233 proteins (27.06%) in ER while the remaining 633 proteins were mainly located in the chloroplast, cytoplasm and nucleus (Table S3, Figure 3A). 

To verify the prediction results of subcellular localization, we selected five representative ER-located DAPs using online prediction to further perform a subcellular localization assay, including protein disulfide isomerase (PDI), α-amylase/trypsin inhibitor (AI/TI), urea glycolate hydrolase (AIIA), GOLD domain-containing protein (TMED2) and signal peptidase complex subunit 2 (Spcs2). We cloned five DAP genes and transformed them into Arabidopsis protoplasts for transient expression, then observed them using confocal microscopes. The ER suborganelle is a relatively large membrane system that connects with the plasma membrane and nucleus and evenly distributes in the cytoplasm. Considering that Arabidopsis protoplast cells are generally small and endoplasmic reticulum subcellular organelles are a large membrane system, the ER proteins may be interfered as connected with other organelle components. We therefore selected ER-specific calreticulin to mark and co-locate the target proteins in Arabidopsis protoplasts, which allowed us to then eliminate the interference of other components. The ER mark protein calreticulin gene carried an RFP fluorescent tag while we cloned the target genes with GFP fluorescent tags that used different and specific primers (Table S4). The results showed that five DAPs were all co-located with ER mark protein calreticulin, verifying their location in ER (Figure 3B). These experimental results are consistent with the website-based prediction results (Figure 3A).

### 2.4. Function Classification of the Salt Stress Responsive DAPs from ER

**Figure 4:**
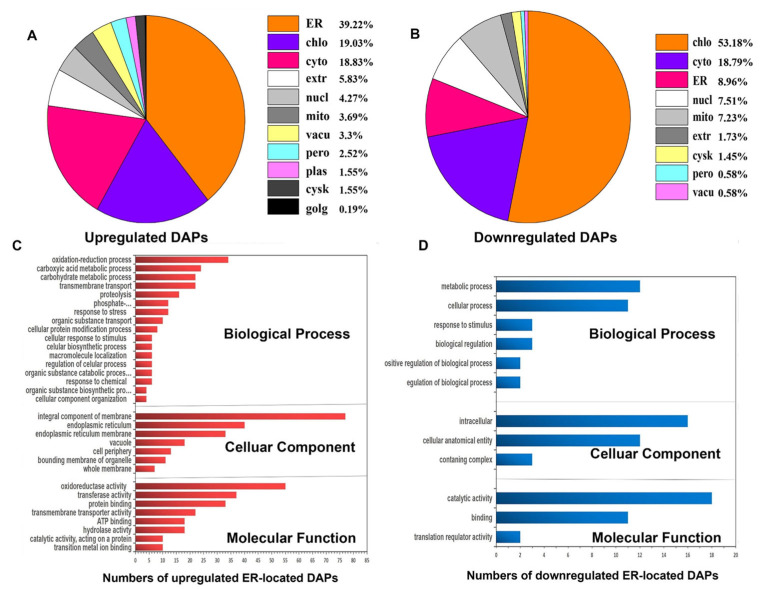
Subcellular localization and functional classification of the identified differentially accumulated proteins (DAPs) in wheat ER under salt stress. (**A**) Subcellular localization of 515 upregulated DAPs identified from wheat ER under salt stress. Chlo, chloroplast; cyto, cytoplasm; mito, mitochondrial; nucl, nucleus; vac, vacuole. (**B**) Subcellular localization of 346 downregulated DAPs identified in wheat ER under salt stress. Chlo, chloroplast; cyto, cytoplasm; mito, mitochondrial; nucl, nucleus; per, peroxisome. (**C**) Functional classification of the ER-localized upregulated DAPs under salt stress. (**D**) Functional classification of the ER-localized downregulated DAPs under salt stress.

Among 515 upregulated and 346 downregulated DAPs induced by salt stress, 202 (39.22%) and 31 (8.96%) were respectively localized in the ER (Figure 4A,B). The function classification of 233 ER DAPs responsive to salt stress was performed using the blast2go software functional annotation. The upregulated ER proteins were mainly involved in organic substance transport, cellular response to stimulus, the phosphate-containing compound metabolic process, stress response, transmembrane transport, the carbohydrate metabolic carboxylic acid metabolic process and the oxidation reduction process (Table S5, Figure 4C). The downregulated ER proteins mainly participated in the metabolic process, biological regulation and the cellular process (Table S5, Figure 4D). Most upregulated ER proteins under salt stress were distributed in ER membrane, as well as ER and an integral component of membrane, which generally had transmembrane transporter activity, protein binding activity, transferase activity and oxidoreductase activity. This indicates that many proteins synthesized in ER play important roles in salt stress response. The downregulated ER proteins primarily exhibit catalytic activity and binding ability in the cellular anatomical entity.

### 2.5. Transcription Expression Analysis of the ER-DAP Genes under Salt Stress 

**Figure 5:**
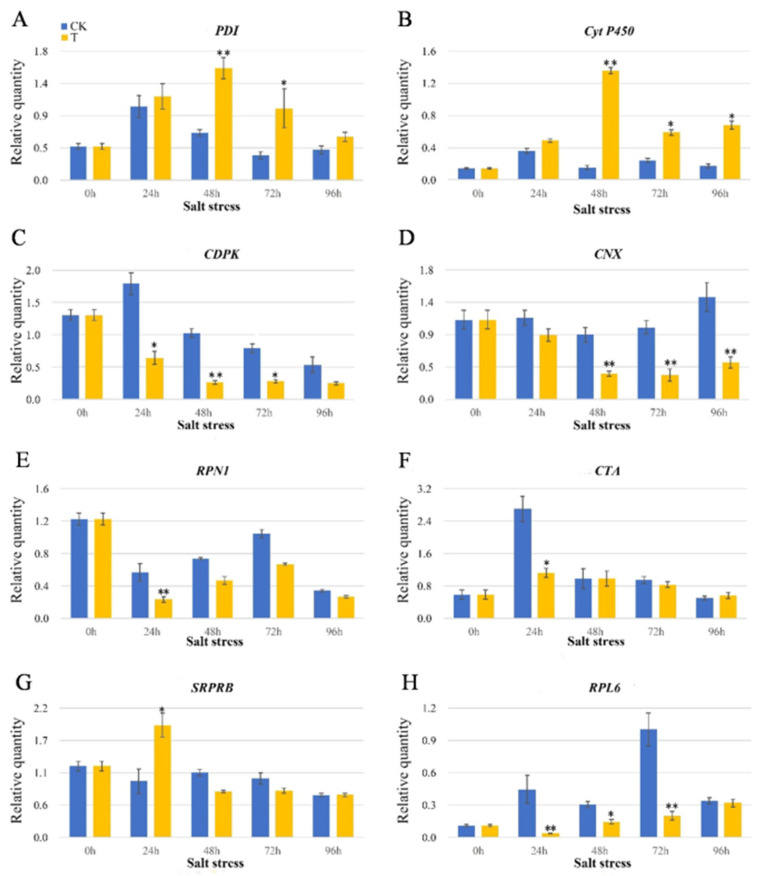
Transcript expression patterns of eight DAP genes in wheat ER under salt stress via RT-qPCR. (**A**) Protein disulfide-isomerase; (**B**) cytochrome P450; (**C**) calcium-dependent protein kinase; (**D**) calnexin homolog; (**E**) dolichyl-diphosphooligosaccharide-protein glycosyltransferase subunit 1 (RPN1); (**F**) calcium-transporting ATPase; (**G**) signal recognition particle receptor subunit beta (SRPRB); (**H**) 50S ribosomal protein L6. Statistically significant differences were calculated based on an independent Student’s *t*-test: * *p* < 0.05; ** *p* < 0.01.

We selected eight DAP genes closely related to ER stress with significant upregulation or downregulation under 0–96 h of salt stress to further detect their dynamic transcriptional expression characteristics under different salt stress treatments. These protein genes included: protein disulfide isomerase (PDI), cytochrome P450 (Cyto P450), calcium-dependent protein kinase (CDPK), calcitonin homologue (CNX), dolichyl-diphosphooligosaccharide-protein glycosyltransferase subunit 1 (RPN1), calcium transport ATP enzyme (CTA), signal recognition particle receptor subunit beta (SRPRB) and 50 s ribosomal L6 protein (RPL6). Table S6 lists the specific primers. As Figure 5 presents, *PDI* and *Cyto P450* genes were significantly upregulated after 48 h of salt stress, while *CTA*, *CNX*, *RPN1*, *CDPK* and *RPL6* genes were significantly downregulated after 24 h and 48 h of salt stress. *SRPRB* showed significant upregulation at 24 h after salt stress, but gradually downregulated after 48 h. Compared to the protein expression patterns, five DAP genes (*PDI*, *Cyto P450*, *CTA*, *RPL6* and *SRPRB*) showed high consistency or similar patterns. The remaining three DAP genes (*CNX*, *RPN1* and *CDPK*) showed poor consistency between transcriptional and translational expression, perhaps because of time-space span between transcription and translation as well as various posttranscriptional and posttranslational modifications [28,29].

## 3. Discussion

Salt stress leads to plants producing and accumulating a large number of ROS, causing oxidative damage to cells [30] and inhibiting plant growth (Figure 1A–D). Meanwhile, a large amount of ROS can also act as a signal of stress response [31,32], inducing the activity increase of plant ROS scavenging-related enzymes such as SOD, CAT and POD (Figure 1F–H). SOD can convert superoxide radicals into oxygen and hydrogen peroxide, and CAT can catalyze the conversion of hydrogen peroxide into water and oxygen. POD can catalyze the oxidation of substrate with hydrogen peroxide as the electron acceptor. These are the key antioxidant enzymes in plant ROS scavenging systems [33]. 

ROS accumulation can cause continuous oxidization and reduction of disulfide bonds [34], which accounts for most of the nicotinamide adenine dinucleotide phosphate (NADPH) in the cell and results in misfolded proteins accumulating. Meanwhile, if unfolded or misfolded proteins accumulate in the ER lumen, the cells face ER stress that induces the unfolded protein response (UPR) to relieve ER stress [35]. The UPR pathway reestablishes ER homeostasis and protein synthesis, including initiating expression of chaperones and foldases for promoting protein folding, attenuating translation and removing unfolded proteins through proteasome degradation [36]. When these misfolded proteins are improperly treated, plant growth inhibition and cell death occur [37]. The current study found that the upregulated ER-DAPs involved in UPR mainly participated in the redox process, cellular stimulus response, phosphate-containing compound metabolic process, stress response and transmembrane transport (Figure 4C). The upregulation of these proteins alleviated the stress pressure of ER under salt stress conditions. 

**Figure 6:**
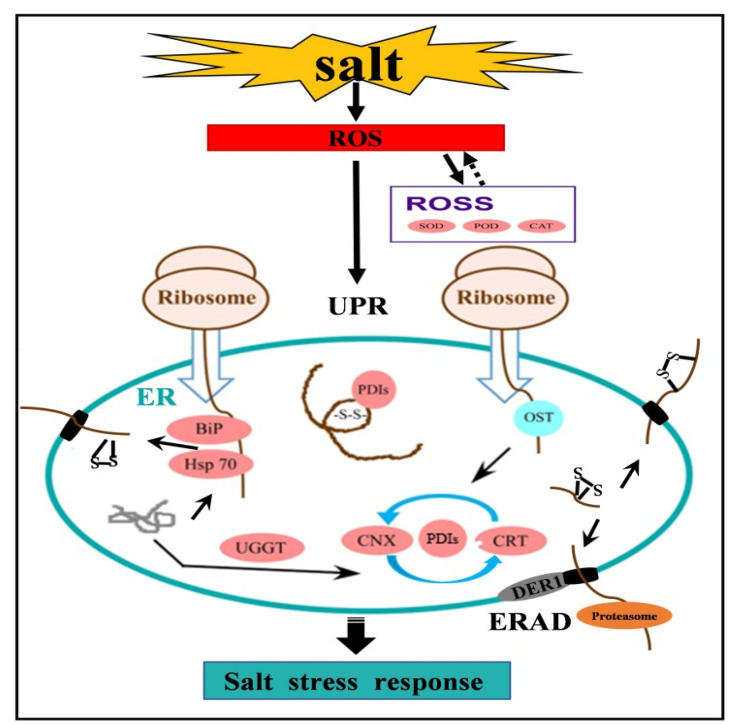
A putative synergistic responsive network of wheat ER proteome to salt stress. The red circle represents upregulated proteins. *BiP*, binding protein; *CAT*, catalase; *CNX*, calnexin; *CRT*, calreticulin; *ERAD*, endoplasmic reticulum associated degradation; *DER1*, degradation in the endoplasmic reticulum protein 1; *Hsp70*, 70 kilodalton heat shock proteins; *ROS*, reactive oxygen species; *ROSS*, reactive oxygen species scavenging; *OST*, oligosaccharide transferase; *PDIs*, protein disulfide-isomerase; *POD*, peroxidase; *SOD*, superoxide dismutase; *UGGT*, UDP-glycosyl transferases; *UPR,* unfolded protein reaction.

ER can anchor the heat shock 40 protein (ERdj3) to form a complex with the molecular chaperone proteins, binding directly to the hydrophobic region of the newborn protein, preventing protein aggregation, and assisting protein folding [38]. We identified two upregulated molecular chaperone proteins (R9W6A6 and A0A3B6QFL1) under salt stress (Table S3). As a kind of subcellular organelle, ER not only participates in maturing and folding protein, but also stores intracellular calcium ions that regulate calcium dependent protease activity in the ER lumen. When calcium ions unbalanced, it affects the folding ability and activity of these proteins, also causing stress response. After calcium ions complete messengers in the cytoplasm, they are repumped into ER storage by calcium-transporting ATPase on the ER. This not only ensures the difference of calcium concentration, but also maintains molecular chaperon proteins’ activity. In a previous report [20], we found that two calcium-transporting ATPases (A0A3B6HQ49 and A0A3B5ZQ67) were significantly upregulated while under salt stress (Table S3, Figure 5). The ER lumen has unique oxidizing potential that supports disulfide bond formation during protein folding, as well as high protein concentration to form a gel-like protein matrix of chaperones and folding enzymes [26]. As a versatile protein folding factory, ER contains a specialized set of folding enzymes including PDI family (A0A3B6IQT3, W5BSJ0 and D8L9B3), PPI (A0A3B6PP75 and A0A3B6TIS2). We detected many such proteins as abundant proteins in the current study (Table S3, Figure 6). 

Another UPR mechanism is mainly involved in attenuating translation as a response to salt stress. Ribosomal proteins play an integral role in generating rRNA structure and forming protein synthesizing machinery. They are also crucial in the growth and development of all organisms [39]. In the current study, we found that the expression of ribosomal related proteins was significantly downregulated such as RPL (A0A1D6BC85), A0A1D5U807, A0A3B5YZT7, 60sRPL (A0A0C4BIR6), A0A3B6C6A1, and A0A3B6RED0 (Table S3). This corresponded to their transcription level (Figure 5). It is possible that this could reduce ER protein concentration under salt stress. Consistent with the previous report on soybeans by [18], protein synthesis was impaired under salt conditions due to the decreased abundance of ribosomal proteins. The slowdown of the protein translation process could help alleviate ER load under salt stress conditions.

The ERAD mechanism is important as a means to remove unfolded proteins in the UPR, but mainly in the CNX/CRT folding cycle. Its basic process primarily includes four steps: target protein recognition, protein ubiquitination, reverse transcriptase transport and proteasome degradation [40]. The intermediate products of protein folding and the final misfolded proteins present a few structural similarities. The hydrophobic region embedded in the protein is exposed to the outside, easily leading to protein aggregation and misfolding. Molecular chaperones such as the heat shock protein 70 family can promote the folding of polypeptide chains by binding to hydrophobic regions. When the target protein interacts with Hsp70, the E3 ubiquitin ligase Hrd1 complex in the ERAD pathway can recognize it [41]. The misfolded proteins were ubiquitinated by E3 ubiquitin ligase on the cytoplasmic sol surface of the ER and then entered 26S proteasome to be degraded. Newborn peptides with glycosylation sites glycosylate when oligosaccharide transferase (OST) enters them. This process transfers pre-combined lipids (polyterpenol)-linked oligosaccharides to glycoprotein-dependent asparagine residues [42]. Monoglycosylated oligosaccharides are recognized by lectin molecular chaperone calcitonin, which is a kind of membrane-anchored protein, but they are also recognized by lumen protein calcium reticulin. These two proteins form a folding cage, thereby folding the target protein. PDI family not only catalyzes the formation of disulfide bonds, but also participates in the CNX/CRT cycle [43]. Moreover, UDP-glucosyltransferase (UGGT) and re-glycosylated detect misfolded proteins to reenter the calcitonin/calreticulin-mediated folding cycle and complete correct folding. UGGT can identify hydrophobic residues clusters exposed on the surface of spherical conformation isomers in the structural domain of unfolded proteins, acting as decision maker in ERQC [43,44,45]. We found that plants significantly upregulated UGGT in response to salt stress (Figure 2I). Several protein family members were also significantly upregulated under salt stress in the ERAD mechanism, including Hsp70 family (A0A3B6GVY6 and A0A3B5XXV7) and calcium (A0A3B6NLQ9 and A0A3B6NMA6) (Table S3). This indicated that the rapid accumulation of misfolded ER proteins could activate the degradation reaction mechanism of ER misfolded proteins under salt stress.

According to our results and previous reports, we proposed a putative metabolic network of wheat ER proteome responsive to salt stress (Figure 6). When plants were subjected to salt stress, ROS was rapidly accumulated. With continuous salt stress, excessive ROS activated the related ROS scavenging (ROSS) to maintain the balance of redox in cells. Meanwhile, ROS promoted the continuous redox of protein disulfide isomerase and accelerated the folding and removal of unfolded and misfolded proteins in the ER. When salt stress continued, excessive accumulation of misfolded proteins in the ER lumen induced significant upregulation of chaperone proteins as a means of reducing the ER load. The upregulation of calcium-transporting ATPasesbalanced the homeostasis of calcium concentration difference, ensured the activity and folding of calcium-dependent proteins and molecular chaperones and reduced the misfolded proteins. The excessive accumulation of misfolded proteins caused ER stress response, resulting in ER-ERAD activation. The unfolded and misfolded proteins accumulated in the ER cavity continue to fold through the cycle of calcitonin and calcium reticulum. Misfolded proteins correctly entered the ER autodegradation system. On the other hand, ER proteins were no longer transported into the ER lumen to relieve ER lumen pressure, and ribosomal proteins greatly reduced, thereby inhibiting the cell process.

## 4. Materials and methods

### 4.1. Wheat Materials and Salt Stress Treatments

Elite Chinese winter wheat cultivar Zhongmai 175 (*Triticum aestivum* L.) was used in this study. The mature seeds with similar size were surfacely sterilizedwith 70% (*v*/*v*) alcohol and 15% (*v*/*v*) sodium hypochlorite and rinsed four times with sterile distilled water. Then, the sterilized seeds were transferred onto wet filter paper and germinated at room temperature. After 48 h, uniformly germinated seeds were picked out and further grown in half strength Hoagland culture solution. The salt stress treatments were applied to wheat seedlings at the three-leaf stage by adding 200 mM NaCl to culture solution. The control group was grown in normal culture solution. Both treatment and control groups included three biological replicates (each with 300 seedlings), and the samples of whole seedlings at 0, 24, 48, 72 and 96 h in salt treatment and control were collected and stored in −80 °C prior to analysis.

### 4.2. Seedling Morphology Observation and Physiological Parameter Measurement

The measurement of plant height and root length were performed on 30 seedlings from the control and treatment group after 0, 24, 48, 72 and 96 h. The relative water content (RWC) and chlorophyll content of wheat seedling leaves were measured based on the method of Lv et al. [46]. The enzyme activities were demonstrated, including catalase (CAT), superoxide dismutase (SOD) and peroxidase (POD) related to reactive oxygen scavenging pathway, using a kit purchased from Suzhou Keming Biotechnology Co., LTD. The activates of UDP-glycosyl transferases (UGGT) and peptidyl-prolyl cis-trans isomerase (PPI) related to ER stress response were demonstrated using an ELISA kit (Jianglai Biological Co., LTD, Shanghai,China) based on the kit instructions. All measurements included three biological replicates to minimize experimental error. Statistical significances of the differences between the control and treatments were determined using a Student’s *t*-test using SPSS 17.0 software (SPSS Institute Ltd., Armonk, NY, USA).

### 4.3. Enrichment of ER Components

In the current study, isolating and purifying ER from wheat seedling leaves was based on the method of Wang et al. [47] with some modifications. Wheat seedling leaves (25 g, about 500 seedlings) from each replicate were collected from each replicate after 48 h of treatment. Fresh leaves were cut into 1 cm pieces and ground using a tissue homogenizer (Ultra Turrax-T18, IKA, Staufen, Germany) with isolation buffer I containing PBS (pH 7.8), 250 mM sucrose, 50 mM HEPES, 5 mM EDTA-2Na, 0.2% caseinhydrolysate, 10% PEG, 0.6% PVPP, 0.2% BSA, 10% glycerin, 5 mM vitamin C tablets, 5 mM DTT, 15 mM CsCl, 0.2 mm PMSF and 1× Protease Inhibitor Cocktail (1 tablet/10 mL; Roche, Basel, Switzerland). The homogenate was filtered through three layers of Miracloth ( Darmstadt, Germany) with two repeats. Then, the filtrate was centrifuged at 200× *g* for 10 min at 4 °C, after which the supernatant was collected and centrifuged at 3000× *g* for 10 min at 4 °C three times. Further, a high-speed centrifugation at 10,000× *g* and 4 °C for 20 min was carried out on the supernatant with two repeats. Then, ultra-high-speed centrifugation with 100,000× *g* at 4 °C for 1 h was conducted immediately, and the precipitate was resuscitated with 6 mL resuspension buffer (PBS [pH 7.8], 250 mM SUR, 50 mM HEPES and 2 mM DTT). Subsequently, the resuspended precipitate was loaded onto discontinuous sucrose gradients (8 mL 21.5% sucrose and 5 mL 37% sucrose) and centrifuged at 65,000× *g* for 30 min at 4 °C in a swing-out rotor. The inner membrane system components containing plasma membrane were collected from 21.5% sucrose gradient. A two-phase separation system (water polymer phase: 40 mL PBS buffer pH 7.8, 2.52 g dextran T-500, 2.52 g polyethylene glycol 4000, 250 mM sucrose, 0.8 M sodium chloride, 0.004 g DTT) was used to separate the inner membrane system and plasma membrane, and the inner membrane system was collected from the lower layer. The ER components were separated from the inner membrane system by a series of discontinuous sucrose gradients (3 mL 2 M sucrose, 5 mL 1.5 M sucrose, 4 mL 1.3 M sucrose, 4 mL 1 M sucrose, 8 mL 0.6 M sucrose), and smooth and rough ER were respectively collected from the interface between 0.6 and 1.3 M sucrose gradients and the precipitation. The mixture of smooth and rough ER were diluted ten times with resuspension buffer and centrifuged at 100,000× *g* for 1 h at 4 °C. The precipitation was immediately used for protein extraction.

### 4.4. Protein Extraction

The extraction of ER proteins was performed based on the previous study [48] with some modifications. Briefly, the ER components were suspended in 7 mL of extraction buffer (0.9 m sucrose, 2% TritonX-100 (*v*/*v*), 0.1 M Tris-HCl, pH 7.5, 50 mm EDTA-2Na, 2% SDS, 1% PVPP, 20 mm DTT, 1 mm PMSF, 1× Protease Inhibitor Cocktail) and ultrasound runs for 2 s and stops for 9 s, which is a cycle. Ultrasound lasts for 10 min with 500 W and the temperature was set as not exceeding 25 °C. An equal volume of Tris-balanced phenol (pH 7.5) was added to the mixture and further completely mixed by grounding in a mortar for 10 min. All the homogenate was transferred to a clean centrifuge tube, centrifuged at 14,000× *g* for 30 min at 4 °C, and the supernatant phenol phase was put into a new tube. Proteins were precipitated by adding four volumes of 100 mM ammonium acetate and remained at −20 °C overnight. On the next day, protein pellets were collected via centrifugations at 14,000× *g* for 30 min and rinsed once with pre-cooling methyl alcohol containing 20 mM *β*-mercaptoethanol, two times with pre-cooling acetone containing 20 mM *β*-mercaptoethanol and finally freeze-dried in a vacuum for subsequent experiments.

### 4.5. Trypsin Digestion and HPLC Fractionation

Sample was sonicated three times on ice using a high intensity ultrasonic processor (Scientz, Ningbo, China) in 500 μL lysis buffer (8 M urea, 1× Protease Inhibitor Cocktail). The remaining debris was removed via centrifugation at 12,000× *g* at 4 °C for 10 min. Finally, the supernatant was collected and the protein concentration was determined with BCA kit [49]. For digestion, the protein solution was reduced with 5 mM DTT for 30 min at 56 °C and alkylated with 11 mM iodoacetamide for 15 min at room temperature in darkness. The protein sample was then diluted by adding 100 mM NH_4_HCO_3_ to urea with a concentration of less than 2 M. Finally, 4 µg trypsin (Promega, Madison, WI, USA) was added to a final enzyme/protein ratio of 1:50 (*w*/*w*) at 37 °C overnight according to a previous study [50]. The tryptic peptides were fractionated into fractions via high pH reverse-phase HPLC using Agilent 300 Extend C18 column (5 μm particles, 4.6 mm ID, 250 mm length). The peptides were gradient eluted with 8‒32% acetonitrile (Na_2_CO_3_-NaHCO_3_ buffer, pH 9) and collected for later analysis.

### 4.6. NanoUPLC and Mass Spectrometric Analysis

The peptides were dissolved in mobile phase A and separated using EASY-nLC 1000 (Thermo/Finnigan, San jose, CA, USA). Mobile phases A and B contained 0.1 % formic acid in water and 0.2% formic acid in 90% acetonitrile, respectively. The gradient was set to 6–23% from 0 to 40 min, 23–35% from 40 to 54 min, 35–80% from 54 to 57 min and 80% from 57 to 60 min, with flow rate setting at 0.40 µL min^−1^. Meanwhile, the LC system was equipped withorbitrap Q Exactive mass spectrometry (Thermo/Finnigan). The *m*/*z* scan range was 350 to 1800 for a full scan, and intact peptides were detected in the Orbitrap at a resolution of 70,000. Automatic gain control (AGC) was set at 5 × 10^4^ Peptides were selected for MS/MS using a NCE setting of 28 and the fragments were detected in the Orbitrap at a resolution of 17,500. The electrospray voltage applied was 2.0 kV.

### 4.7. Sequence Database Search and Data Analysis

Maxquant (v1.5.2.8 Max Planck Institute for Biochemistry, Martinsried, Germany) was used to complete the retrieval of secondary mass spectrometry data from UniProt *Triticum aestivum* containing 116,790 sequences. The method of trypsin digestion was set to the maximum number of modified peptides to 5 and the minimum length to 7 amino acid residues, the parameter of omission site to 2, and the mass tolerance for precursor ions was set as 20 ppm in the first search and 5 ppm in the main search, and the mass tolerance for fragment ions was set as 0.02 Da. The fixed modification was set to cysteine alkylation, the oxidation of methionine and the acetylation of protein N-terminal were set to variable modification, and the false positive rates of protein identification was set to 1% [51,52]. In this study, the quantitative values of each sample in three replicates were obtained via LFQ intensity. The ratio of the mean LFQ intensity between the two samples represents the protein fold change. To calculate the significant *p* value of differential expression between two samples, LFQ intensity was taken as log2 transform. Then, when the protein is quantified at least twice in the two compared samples, the *p* value is calculated using the two-tailed *t* test. When the *p* value < 0.05 and protein ratio > 2, it was regarded as up-regulation. When the *p* value < 0.05 and protein ratio < 0.5, it was regarded as down-regulation. (Student’s *t*-test, *p* < 0.05).

### 4.8. Western Blotting

For immunoblotting analysis, approximately 10 µg of total proteins and chloroplast proteins were prepared and separated using 12% sodium dodecyl sulfate-polyacrylamide gel electrophoresis (SDS-PAGE). The separated proteins on the gel were transferred to polyvinylidene fluoride (PVDF) membrane via semi-dry transfer imprinting and incubated in blocking buffer containing 20 mM of trihydrochloric acid (pH 7.5), 500 mM of sodium chloride and 5% skim milk, and then further incubated with 1 RV 5000 diluted polyclonal antibody (Swedish Agrisera, Vännäs, Sweden) at room temperature for 1 h. Anti-rabbit or mouse antibodies (Bio-Rad, Hercules, CA, USA) conjugated with horseradish peroxidase or anti-mouse antibodies (Bio-Rad) conjugated with horseradish peroxidase were used as secondary antibodies. After incubating with the second antibody for 1 h, the signals were detected using an ECL plus Western blotting kit (General Electric Healthcare, Piscataway, NJ, USA) according to the manufacturer’s instruction.

### 4.9. Subcellular Localization

The subcellular localization predication of the identified proteins were performed according to the combination of the predicated results from WoLF PSORT (https://wolfpsort.hgc.jp/, accessed on 28 June 2020), TargetP-2.0 (http://www.cbs.dtu.dk/services/TargetP/, accessed on 28 June 2020), Plant-mPLoc (http://www.csbio.sjtu.edu.cn/bioinf/plant-multi/, accessed on 29 June 2020), CELLO (http://cello. life.nctu.edu.tw/, accessed on 29 June 2020) and UniProtKB (https://www.uniprot.org/help/uniprotkb/, accessed on 29 June 2020). Then, further subcellular localization assay by transforming Arabidopsis protoplasts was performed to verify the predicated results according to [53]. The amplified target fragment was reconstructed onto pSAT1-GFP-N (Pe3449) and PSAT1-RFP-N (Pe3449) vectors. Psat1-gfp-n (Pe3449) and PSAT1-YFP-N (Pe3449) carried a green fluorescent protein (GFP) gene and a red fluorescent protein (RFP) gene, respectively.

### 4.10. Total mRNA Extraction and Real-Time Quantitative Polymerase Chain Reaction (RT-qPCR)

RT-qPCR was used to detect the dynamic transcript levels of the key DAPs genes in response to salt stress. Total RNA was isolated from seedling leaves of control and salt treatment groups using TRIZOL Reagent (Invitrogen, Carlsbad, CA, USA). Genomic DNA was removed and then the reverse transcription reactions were performed using a PrimeScript^®^ RT Reagent Kit with gDNA Eraser (TaKaRa, Shiga, Japan) according to [54]. Gene-specific primers for selected genes were designed using online Primer3Plus (www.bioinformatics.nl/cgi-bin/primer3plus/primer3plus.cgi). Ubiquitin was used as the reference for normalization. RT-qPCR was conducted using a CFX96 Real-Time PCRD detection system (Bio-Rad), and all data were analyzed with CFX Manager Software (Bio-Rad). Three independent replications were conducted for each sample.

## 5. Conclusions

Salt stress significantly inhibited the growth of wheat seedlings and resulted in phenotypic, physiological and biochemical changes, including the decrease of plant height, root length, relative water content and chlorophyll content. Label-free quantitative proteomic analysis identified 234 ER-localized DAPs in response to salt stress, including 203 upregulated and 31 downregulated proteins. The upregulated proteins were mainly involved in protein folding and quality control, ER stress response, unfolded protein response and ER-related degradation, while the downregulated proteins mainly participated in basic plant metabolic processes such as protein synthesis and translation. Through two main pathways of UPR and ERAD regulating ER stress in plants, the synergistic response of these ER proteins could play important roles in plant salt stress defense.

## Supplementary Materials

The following are available online at https://www.mdpi.com/article/10.3390/ijms22094840/s1, Table S1: The list of peptides identified in wheat endoplasmic reticulum under salt stresses, Table S2: The list of total proteins identified in wheat endoplasmic reticulum under salt stresses, Table S3: The list of differentially accumulated proteins (DAPs) identified from extracted endoplasmic reticulum of wheat seedling leaves under salt stress, Table S4: List of specific primers for gene cloning and vector construction, Table S5: GO annotation of ER-localized differentially accumulated proteins (DAPs) identified in wheat endoplasmic reticulum under salt stresses, Table S6: List of specific primers for RT-qPCR analysis.

## Abbreviation

AGPase	ADP glucose pyrophosphorylase
AI/TI	Alpha-amylase/trypsin inhibitor
AIIA	Probable ureidoglycolate hydrolase
AOX1/2	Plant alternative oxidase 1 and 2
BiP	Binding protein
CAT	Catalase
CNX	Calnexin
CDPK	Calcium-dependent protein kinase
CRT	Calreticulin
CTAB	Hexadecyl trimethyl ammonium bromide
DAPs	Differentially accumulated proteins
DPA	Day post anthesis
ER	Endoplasmic reticulum
ERAD	Endoplasmic reticulum associated degradation
ERQC	Endoplasmic reticulum quality control
GO	Gene ontology
H3	Histones 3
HSP	Heat shock protein
NADPH	Nicotinamide adenine dinucleotide phosphate
OST	Oligosaccharide transferase
PDI	Protein disulfide isomerase
PEG	Polyethyleneglycol
PMSF	Phenylmethylsulfonyl fluoride
POD	Peroxidase
PPI	Peptidyl-prolyl cis-trans isomerase
RT-qPCR	Real-time quantitative polymerase chain reaction
RFP	Red fluorescent protein
ROS	Reactive oxygen species
ROSS	Reactive oxygen species scavenging
RP	Ribosomal proteins
RP-HPLC	Reversed-phase high performance liquid chromatogram
RT-PCR	Reverse transcription polymerase chain reaction
RWC	Relative water content
SDS	Sodium dodecyl sulfate
SDS-PAGE	SDS-polyacrylamide gel electrophoresis
SOD	Superoxide dismutase
SOS	Salt overly sensitive
Spcs2	Probable signal peptidase complex subunit 2
UGGT	UDP-glycosyl transferase
UPR	Unfolded protein reaction
